# Effect of 1,25-Dihydroxyvitamin D3 on Stem Cells from Human Apical Papilla: Adhesion, Spreading, Proliferation, and Osteogenic Differentiation

**DOI:** 10.1155/2021/1481215

**Published:** 2021-10-08

**Authors:** Yonggang Ma, Jiaojiao Yang, Yan Li, Lijie Sun, Zhuyun Liu, Jia Qian, Lizhong Wang, Delin Xia

**Affiliations:** ^1^Department of Oral and Maxillofacial Surgery, Affiliated Stomatological Hospital of Southwest Medical University, Luzhou 646000, Sichuan Province, China; ^2^Taizhou Polytechnic College, School of Pharmacy, Bone Tissue Engineering Research Center of Taizhou, Taizhou, Jiangsu 225300, China; ^3^Plastic and Maxillofacial Surgery Department, Second Affiliated Hospital of Chongqing Medical University, Chongqing 400010, China

## Abstract

Currently, it still remains a difficult problem to treat apical insufficiency of young permanent teeth resulted from pulp necrosis or periapical periodontitis. Previous studies have demonstrated that the treatment of revascularization using stem cells from apical papilla (SCAPs) results in increased root length and thickness of traumatized immature teeth and necrotic pulp. In this study, we investigated the role of 1,25-dihydroxyvitamin D3 in regulating the adhesion, spreading, proliferation, and osteogenic differentiation of SCAP, laying the foundation for subsequent clinical drug development. The immature tooth samples were collected in clinical treatment. SCAPs with stable passage ability were isolated and cultured. The multidifferentiation potential was determined by directed induction culture, while the stem cell characteristics were identified by flow cytometry. There were three groups: group A—SCAPs general culture group; group B—SCAPs osteogenesis induction culture group; and group C—SCAPs osteogenesis induction culture+1,25-dihydroxyvitamin D_3_ group, and the groups were compared statistically. The proliferation of SCAPs in each groups was detected through CCK-8 assay. RT-qPCR was used to detect the transcription levels of *Runx2*, *ALP*, *Col I*, and *OCN* of SCAPs in each groups. Results exhibited that the isolated SCAPs had multidifferentiation potential and stem cell characteristics. After 24 h culturing, cells in group C spread better than those in groups A and B. The proliferation activity of SCAPs factored by CCK-8 ranked as group C > group B > group A, while the transcription levels of Runx2, ALP, Col I, and OCN leveled as group C > group B > group A. These results suggested that 1,25-dihydroxyvitamin D_3_ can significantly promote the adhesion, spreading, and proliferation of SACPs and improve the osteogenic differentiation of SCAPs by means of regulating upward the transcription level of osteogenic differentiation marker.

## 1. Introduction

Among preschool children, school-age children, and young people, traumatic tooth injury (TDI) occurs very frequently, accounting for about 5% of all trauma people seek treatment for [1]. According to a 12-year literature review report, 25% of all school children experience dental trauma, while 33% of adults suffered permanent dentition trauma, with the majority of injuries occurring before age nineteen [2]. Since the roots of young permanent teeth have not yet developed, young permanent tooth pulp disease or periapical disease caused by caries and tooth trauma will cause their roots to stop developing, eventually resulting in shortened tooth roots, weak root canal walls, and root problems such as reduced pressure resistance and incomplete apical hole closure [3]. It will seriously affect the service life of permanent teeth and the establishment of occlusal relationship, which inevitably exert many adverse effects on the pronunciation and coordination abilities of young children in their later stages of growth. In recent years, Banchs and Trope [4] had proposed a new type of treatment called “Dental Pulp Revascularization,” which is aimed at the incomplete apical closure caused by pulp necrosis or apical periodontitis of young permanent teeth that have not yet formed roots. Studies have showcased that the success of this treatment plan is closely related to stem cells derived from periapical tissues [5, 6].

As a type of cells isolated from papillary tissues of apical teeth, stem cells from apical papilla (SCAPs) possess significant proliferation capabilities, multidirectional differentiation potential, and self-renewal ability. SCAPs are a type of odontogenic mesenchymal stem cells, which are likely to be precursor cells that form dentin cells, and can induce the differentiation of dentin cells and promote the regeneration of dental pulp and the increased root length and thickness of traumatized immature teeth and necrotic pulp [7, 8].

1,25-Dihydroxyvitamin D_3_ is an important hormone regulating calcium and phosphorus metabolism in the human body and also a key regulator of cell growth and differentiation, immune system function, and central nervous system function [9]. Studies have shown that adding 1,25-dihydroxyvitamin D_3_ to the osteogenic induction medium can significantly promote the osteogenic differentiation of induced multifunctional stem cells and mesenchymal stem cells [10, 11]. However, the mechanism of 1,25-dihydroxyvitamin D_3_ on osteogenic differentiation of stem cells from apical papilla functions is still unclear.

In this study, we isolated and purified the SCAPs; then, the 1,25-dihydroxyvitamin D_3_ was used to intervene in SCAPs in vitro to investigate the role of 1,25-dihydroxyvitamin D3 in regulating the adhesion, spreading, proliferation, and osteogenic differentiation of SCAPs, laying the foundation for subsequent clinical drug development.

## 2. Materials and Methods

### 2.1. Materials

The following are the materials used in the study: DMEM medium (Hyclone, USA); fetal bovine serum (Sijiqing Company, China); penicillin-mixed solution (North China Pharmaceutical, China); pancreatin digestion solution (Gibco, USA); PBS buffer (Beyotime, China); Oil Red O stain kit (Solarbio, China); alkaline phosphatase staining kit (Beyotime, China); Alizarin Red S staining kit (Solarbio, China); mouse anti-human STRO-1-FITC, CD45-FITC, CD90-FITC, and CD146-FITC monoclonal antibodies (eBioscience, USA); tetramethyl azo thiazole salt (MTT); 4′6-diamidino-2 phenylindole dihydrochloride aqueous solution dihydrochloride aqueous solution (DAPI solution) (Dojindo, Japan); rhodamine marked phalloidin (Cytoskeleton, USA); paraformaldehyde (Merck, Germany); Triton X-100 (Sigma-Aldrich, USA); 3-isobutyl-1-Methylxanthine (IBMX) (Beyotime China); *β*-glycerophosphate sodium (North China Pharmaceutical, China); insulin (North China Pharmaceutical, China); indomethacin (North China Pharmaceutical, China); dexamethasone (North China Pharmaceutical, China); TRIZOL reagent (Invitrogen, USA); Revert Aid First Strand cDNA Synthesis Kit (Thermo Fisher Scientific, USA); SYBR Green PCR Master Mix (Toyobo Life Science, Japan); and total RNA extraction kit (spin column type, Tiangen Biochemical, China). Samples of young permanent teeth are obtained from healthy premolars or third molars being removed due to orthodontic treatment or wisdom tooth impaction at the age of 12-18 years (the patient's informed consent).

### 2.2. Methods

#### 2.2.1. Isolation and Cultivation of SCAPs

The isolation and purification protocol of SCAPs are as described in the previous study [12]. Six premolars or third molars of patients with orthodontic treatment needs were collected from the Affiliated Dental Clinic of Taizhou Polytechnic College, and the experimental protocols in this study were approved by the Ethics Committee for Research of Taizhou Polytechnic College. Sample collection requirements were as follows: the patient was 12-18 years old, with normal tooth development, healthy tooth tissue, and no dental caries and periapical disease. Before tooth extraction, the patient was instructed to gargle 3% hydrogen peroxide for 1 min. The extracted teeth were immediately placed in the preprepared DMEM complete medium containing 3% penicillin-streptomycin, and the sample processing time does not exceed 2 hours. The sterile scissors were used to cut out approximately 0.2 × 0.2 cm^2^ apical papillary tissue. After centrifuging to remove the supernatant, 2 mL of digestion solution (3 mg/mL type I collagenase solution and 4 mg/mL dispose enzyme solution) was added to digest the apical papillary tissue for 30 min. After immersion in PBS, culture medium was added to prepare cell suspension. The cell suspension was evenly inoculated into the cell culture flask and placed in a 37°C constant temperature incubator to continue culturing. After 7-10 days of culture, the cells can be observed crawling out under the microscope. Change the medium every other day, and culture it until the cells are 80% confluent, and then passage them at the ratio of 1 : 2.

#### 2.2.2. Identification of SCAPs

*(1) Flow Cytometry.* The third-generation CAPs were diluted with PBS solution to prepare a suspension with a cell density of 1 × 10^6^ cells/mL, and 100 microliters of the cell suspension was added to four different centrifuge tubes. 0.5 *μ*g of STRO-1-FITC, 0.25 *μ*g of CD146-FITC, 1 *μ*g of CD90-FITC, CD45-FITC, and 0.5 *μ*g of CD90-FITC were added to the above centrifuge tube and incubated for 30 minutes in a refrigerator at 4°C in the dark. The cell suspension prepared in PBS solution was used as a blank control, and the positive expression rate of SCAPs-specific surface antigen markers STRO-1, CD146, and CD90 and the negative expression rate of CD45 were detected by flow cytometry.

*(2) Directed Differentiation Induction*.

- Osteogenesis induction: after the third generation, SCAPs proliferated and fused to 80%; osteoinduction medium (containing 10 mM *β*-glycerophosphate, 50 *μ*g/mL ascorbic acid, and 10-8 M dexamethasone) was added for osteoinduction culture. After culturing for 14 days, the medium was aspirated, and 4% paraformaldehyde was added to the cells and fixed at 4°C in the dark. The alkaline phosphatase kit was used for ALP staining to detect the early osteogenic differentiation performance of SCAPs. After 21 days of osteogenic induction culture, the SCAPs were fixed with 4% paraformaldehyde at 4°C in the dark. The Alizarin Red S staining kit was used to detect the osteogenic mineralization of SCAPs.
- Adipogenesis induction: when the third passage SCAPs grew and fused to 80%, the adipogenic differentiation medium (containing 10^−7^ mol/L dexamethasone, 10 mg/mL insulin, 0.2 mmol/L indomethacin, and 0.5 mmol/L 3-isobutyl-1-methylxanthine) was added for adipogenic induction culture. After culturing for 21 days, the SCAPs were fixed with 4% paraformaldehyde at 4° C in the dark. The Oil Red O kit was used to stain cell lipids to reflect the adipogenic differentiation potential of SCAPs.

#### 2.2.3. Experimental Grouping

Take and inoculate the 3rd passage SCAPs into 6-well plates at 1 × 10^6^ cells/well. The experiment was categorized into three groups: group A as the general culture group of SCAPs, group B as the osteogenic induction culture group of SCAPs, and group C as the bone formation of SCAPs induction culture and 1,25-dihydroxyvitamin D_3_ group. Groups B and C were cultured with osteogenic induction medium, whereas group C was added with 10 nmol/L 1,25-dihydroxyvitamin D_3_ [7].

#### 2.2.4. The Effect of 1,25-Dihydroxyvitamin D_3_ on the Adhesion and Spread of SCAPs

After SCAPs were cultured with 1,25-dihydroxyvitamin D_3_ in a 37°C constant temperature incubator for 24 h, the medium was aspirated and the cells were fixed at 4°C with paraformaldehyde. After immersion in PBS buffer, 0.25% Triton X-100 was used to permeate the cells at room temperature for 10 min of PBS immersion. Add Rhodamine phalloidin staining solution; incubate at 4°C overnight; and discard staining solution with 3 times' PBS immersion, 5 min each time. Then, add DAPI staining solution, and stain at room temperature for 5 min with 3 times' PBS immersion. Observe and take pictures with a laser confocal microscope [13].

#### 2.2.5. The Effect of 1,25-Dihydroxyvitamin D_3_ on the Proliferation of SCAPs by CCK-8

Use the 3rd passaged SCAPs to inoculate 96-well cell culture plates at the density of 4 × 10^3^ cells/well. Set up each experimental group with 6 replicate wells, and repeat the test 3 times. Terminate the cultivation on the 1st, 3rd, 5th, and 7th days after the cultivation. Add 20 *μ*L CCK-8 working solution in the dark (CCK‐8 stock solution/complete medium = 1/10 (*v*/*v*)) to each well to be tested, and incubate for 2 hours in the dark. The absorbance (OD) value was detected through a microplate reader at a wavelength of 490 nm.

#### 2.2.6. RT-qPCR Detection of Transcription Levels of Osteogenic Differentiation Markers

Using 1,25-dihydroxyvitamin D_3_ to intervene and culture SCAPs to day 7 and day 14, then use the total RNA extraction kit to extract total RNA in the sample. Detect RNA concentration with a NanoDrop spectrophotometer. According to the RNA concentration, the template cDNA was obtained using a reverse transcription kit and diluted 10 times with DEPC water. Use SYBR green as a fluorescent dye, and use a real-time fluorescence quantitative PCR instrument to perform PCR amplification reaction in a 10 *μ*L reaction system. The amplification procedure was as follows: 95°C for 10 min, 95°C for 10 min, 55°C for 15 s, and 72°C extends for 15 s for a total of 40 cycles. The housekeeping gene GAPDH is adopted as an internal reference, normalizing the detection results by calculating the relative quantitative *ΔΔ*CT fluorescence.  Table 1 illustrates the sequences of the osteogenic-related genes detected in the experiment.

#### 2.2.7. Statistical Analysis

The experimental data was expressed as the mean ± standard deviation, and the statistical software SPSS20.0 was used for one-way ANOVA (*α*: 0.05).

## 3. Results

### 3.1. Isolation, Cultivation, and Identification of SCAPs

**Figure 1:**
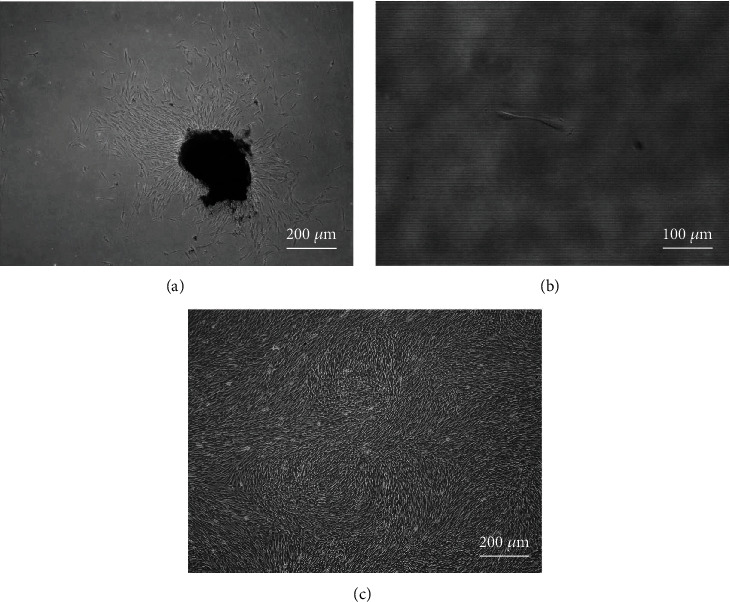
Culture of stem cells from apical papilla. (a) SCAPs cell primary extraction, ×40; (b) SCAPs cell clone, ×400; (c) SCAPs cell proliferation morphology, ×40.

In this experiment, the primary SCAPs were isolated by enzyme digestion. When the apical papilla tissue block was cultured up until the fifth day, morphologically irregular, long fusiform, and small volume cells were seen to crawl out from the edge of the tissue block under an inverted microscope. Then, after 2 weeks of culture, those cells can be observed to grow into normal morphology, achieve good growth status, and develop in a spiral arrangement (Figure 1(a)). Primary SCAPs were digested with 0.25% trypsin and inoculated into 96-well culture plates after centrifugation. After 2 day of culture, a single cell could be seen to proliferate and divide into two cells under an inverted microscope, indicating mitotic ability of the acquired (Figure 1(b)). Then, the primary SCAPs cells progressed to the second generation, with more uniform and fusiform cell morphology at a radial or swirling arrangement when observed under an inverted microscope (Figure 1(c)).

**Figure 2:**
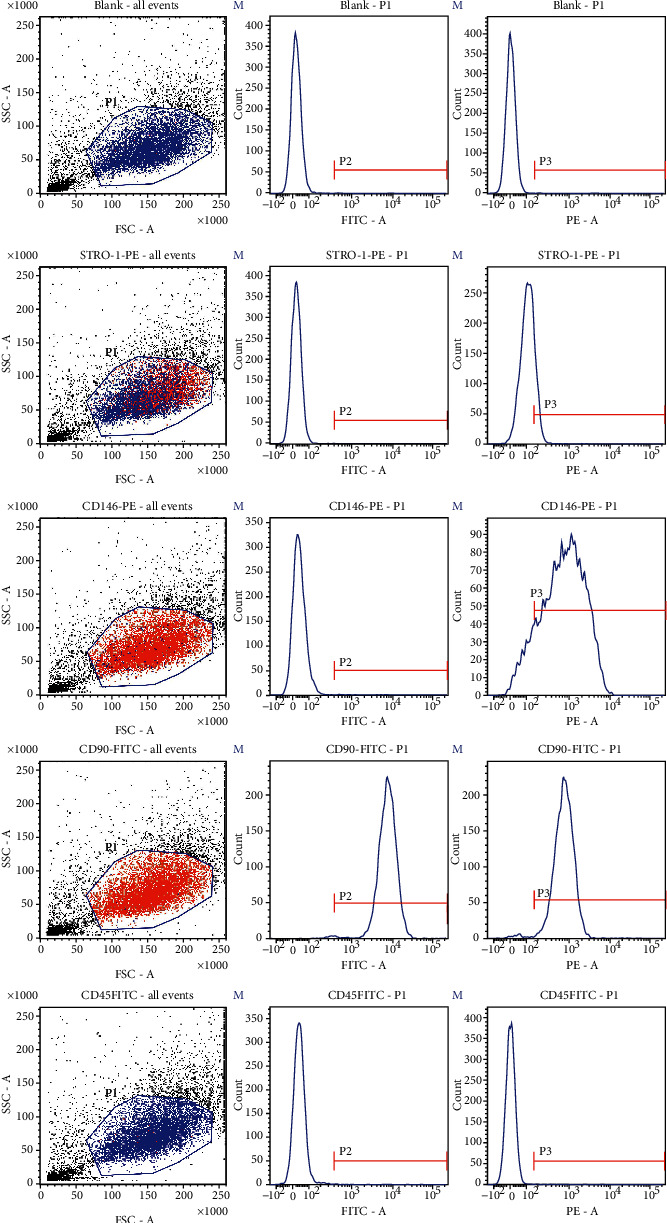
Flow cytometry identification of SCAPs surface antigen. The SCAPs we isolated positively expressed mesenchymal stem cell markers, such as STRO-1 (%parent was 20.97% and %Total was 14.73%), CD146 (%parent was 88.66% and %Total was 61.63%), and CD90 (%parent was 99.20% and %Total was 65.45%), while negatively expressing hematopoietic cell markers, such as CD45 (%parent was 0.29% and %Total was 0.19%).

The results of flow cytometry showed that the SCAPs we isolated positively expressed mesenchymal stem cell markers, such as STRO-1 (%parent was 20.97% and %Total was 14.73%), CD146 (%parent was 88.66% and %Total was 61.63%), and CD90 (%parent was 99.20% and %Total was 65.45%), while negatively expressing hematopoietic cell markers, such as CD45 (%parent was 0.29% and %Total was 0.19%), indicating that the SCAPs isolated in this experiment have the characteristics of mesenchymal stem cells [14] (Figure 2).

**Figure 3:**
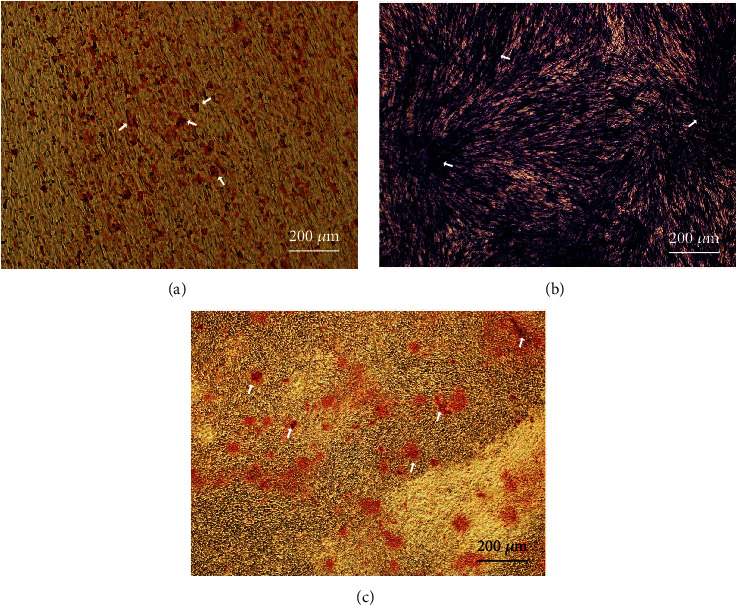
Identification of human root tip papillary stem cells. (a) Oil red O staining after 21 days of SCAPs-induced differentiation, lipid droplets indicated by arrows, ×100; (b) ALP staining of SCAPs-induced differentiation for 14 days, ALP staining-positive areas indicated by arrows, ×100; (c) Alizarin Red staining after 21 days of SCAPs-induced differentiation, mineralized nodules indicated by arrows, ×100.

After 21 days of in vitro adipogenic differentiation, use Oil Red O to stain SCAPs and observe under an inverted microscope that the cell morphology became round and enlarged and that the lipid droplets in the cell cytoplasm were stained red, unevenly distributed, and of different sizes (Figure 3(a)). SCAPs were stained with ALP after 14 days of osteogenic induction culture in vitro, and afterwards, the cells were blue-violet positively stained under an inverted microscope (Figure 3(b)). Induce SCAPs to differentiate in osteogenic induction fluid for 21 days, and then stain it with Alizarin Red. The bottom of the cell culture dish was red to the naked eye, the SCAPs grew vigorously and stratified under the microscope, and the extracellular matrix exhibited reddish brown calcium node (Figure 3(c)).

### 3.2. Effect of 1,25-Dihydroxyvitamin D_3_ on Adhesion and Spreading of SCAPs

**Figure 4:**
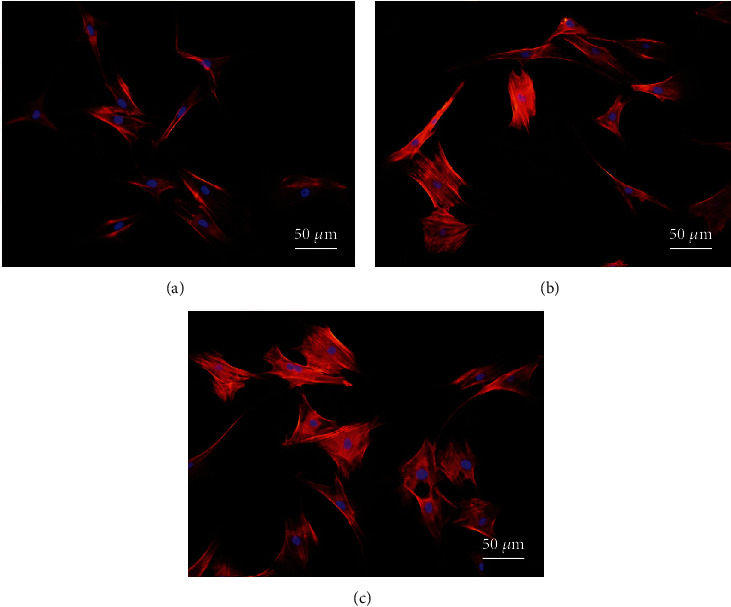
Spreading of SCAPs after 24 hours of cultivation. Immunofluorescence staining: red is F-actin and blue is cell nucleus, ×400; group A: common medium group; group B: osteogenic induction culture group; group C: osteogenic induction culture+1,25-dihydroxyvitamin D_3_ group.

**Figure 5:**
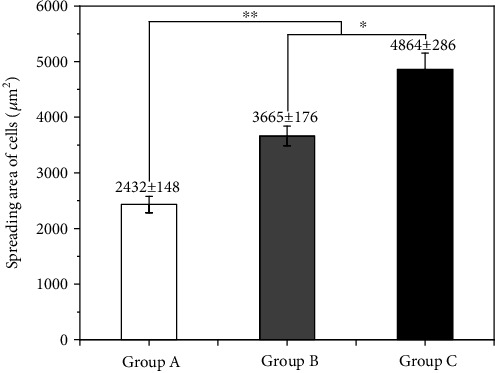
Cell spreading areas (*n* = 200) after seeding of 24 h (∗ means *p* < 0.05; ∗∗ means *p* < 0.01). Comparison of the cell adhesion spreading area was as follows: group C > group B > group A. Through the calculation of Image-Pro Plus software, the cell spreading area in group C (4864 ± 286 *μ*m^2^) was larger than those in group B (3665 ± 176 *μ*m^2^) and group C (2432 ± 1148 *μ*m^2^).

The normal assembly of the cytoskeleton proves to be of great significance to cellular proliferation and differentiation in the later stage. Therefore, this experiment investigated the effect of 1,25-dihydroxyvitamin D_3_ on the adhesion and spreading behavior of SCAPs through the F-actin staining results of SCAPs skeleton. Results demonstrated that the cell adhesion and spreading of SCAPs induced by 1,25-dihydroxyvitamin D_3_ were significantly better enhanced than those of the other two groups with better indicators such as more F-actin assembly, more regular distribution, and significantly larger spreading area (Figure 4). However, the SCAPs spreading and cytoskeletal assembly in the general culture group were inferior to the experimental group. Comparison of the cell adhesion spreading area was as follows: group C > group B > group A. Through the calculation of Image-Pro Plus software, the cells spreading area in group C (4864 ± 286 *μ*m^2^) was larger than those in group B (3665 ± 176 *μ*m^2^) and group A (2432 ± 1148 *μ*m^2^) (Figure 5).

### 3.3. Effects of 1,25-Dihydroxyvitamin D_3_ on the Proliferation of SCAPs

**Figure 6:**
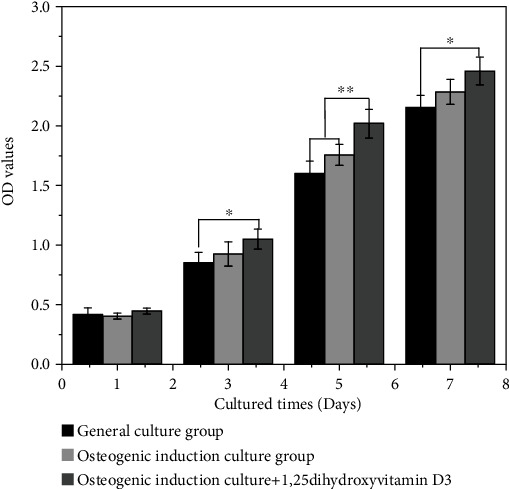
SCAPs cell proliferation activity; ∗ indicates *p* < 0.05. The results of CCK-8 detecting the effect of 1,25-dihydroxyvitamin D_3_ on the proliferation activity of SCAPs are shown. It seemed that no significant difference in the OD values existed on the first day between the control and experimental groups (*p* > 0.05). From the 3rd day, the OD value of the osteogenic induction culture+1,25-dihydroxyvitamin D_3_ group was considerably higher than that of the normal medium group (*p* < 0.05). On the 5th day, the cell proliferation activity of the osteogenic induction culture+1,25-dihydroxyvitamin D_3_ group was immensely better than that of the ordinary medium group and the osteogenic induction culture group (*p* < 0.05). On the 7th day, this trend was further expanded, inferring that the 1,25-dihydroxyvitamin D_3_ group can greatly promote SCAPs proliferation.

The results of CCK-8 detecting the effect of 1,25-dihydroxyvitamin D_3_ on the proliferation activity of SCAPs are shown in Figure 6. It seemed that no significant difference in the OD values existed on the first day between the control and experimental groups (*p* > 0.05). Nonetheless, from the 3rd day, the OD value of the osteogenic induction culture+1,25-dihydroxyvitamin D_3_ group was considerably higher than that of the normal medium group (*p* < 0.05). Furthermore, on the 5th day, the cell proliferation activity of the osteogenic induction culture+1,25-dihydroxyvitamin D_3_ group was immensely better than those of the ordinary medium group and the osteogenic induction culture group (*p* < 0.05). What is more, and on the 7th day, this trend was further expanded.

### 3.4. Detection of Transcription Levels of Osteogenic Differentiation Markers in SCAPs

**Figure 7:**
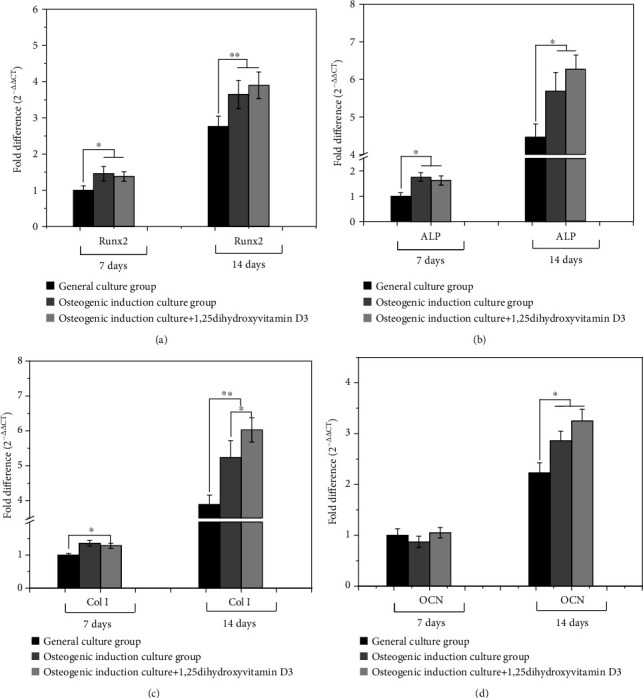
Gene transcription levels of Runx2 (a), ALP (b), Col I (c), and OCN (d) after 7 and 14 days of SCAPs treatment (^∗^*p* < 0.05, ^∗∗^*p* < 0.01). After three different treatments of SCAPs to 7 days, the transcription levels of Runx2, ALP, and Col I in the osteogenic induction culture group intervened by 1,25-dihydroxyvitamin D_3_ were significantly higher than those in the normal culture group, but there was no significant difference of the OCN transcription level in each group. Up until the 14th day of culture, the transcription levels of Runx2, ALP, Col I, and OCN in each group vastly increased, showing that the osteogenic induction culture group and 1,25-dihydroxyvitamin D_3_ intervention group were still higher than the normal culture group with 1,25-dihydroxyvitamin D_3_ intervention group as the highest transcription level.

The RT-qPCR results (Figure 7) showed that after three different treatments of SCAPs to 7 days, the transcription levels of Runx2, ALP, and Col I in the osteogenic induction culture group intervened by 1,25-dihydroxyvitamin D_3_ were significantly higher than those in the normal culture group, but there was no significant difference of the OCN transcription level in each group. Up until the 14th day of culture, the transcription levels of Runx2, ALP, Col I, and OCN in each group vastly increased, showing that the osteogenic induction culture group and 1,25-dihydroxyvitamin D_3_ intervention group were still higher than the normal culture group with 1,25-dihydroxyvitamin D_3_ intervention group as the highest transcription level.

## 4. Discussion

### 4.1. Identification of Stem Cell Characteristics of SCAPs

A previous study reported that stem cells from apical papilla (SCAPs) were like other populations of mesenchymal stem cells exhibiting characteristic features of stem/progenitor cells including the potential for multilineage differentiation and self-renewal [15]. Therefore, in this experiment, four surface markers such as STRO-1, CD146, CD90, and CD45 were selected to detect the stem cell characteristics of the obtained cells. The results showed that the high expression of STRO-1, CD146, and CD90-positive rate and the low expression of CD45-negative rate confirmed the fact that SCAPs are stem cells of mesenchymal origin. In addition, the SCAPs obtained in this study can be differentiated into osteoblasts and adipocytes by induction culture in vitro and have the potential for multidirectional differentiation. Therefore, the cells obtained by means in this study are identified as mesenchymal stem cells, which possess the potential of multidirectional differentiation under a suitable induction environment in vitro.

In addition, it is reported that SCAPs expanded in vitro can differentiate into functional dentin cells to regenerate typical dentin-like structures [16]. Moreover, SCAPs can also regenerate vascularized human dental pulp-like tissue after implantation in empty human root canals, which suggests that SCAPs may be a suitable source of odontoblast progenitor cells, and are responsible for the formation of root dentin [15]. However, there is limited information regarding how SCAPs respond to 1,25-dihydroxyvitamin D_3_; this understanding is key to their potential clinical application.

### 4.2. Effects of 1,25-Dihydroxyvitamin D_3_ on Adhesion, Spreading, Proliferation, and Osteogenic Differentiation of SCAPs

The results of immunofluorescence staining cytoskeleton displayed that 10 nmol/L 1,25-dihydroxyvitamin D_3_ could promote the adhesion and spreading of SCAPs cells at the early stage of inoculation. In the meantime, the CCK-8 test indicated that 1,25-dihydroxyvitamin D_3_ could significantly promote SCAPs, namely, better proliferation than the osteogenic induction medium.

The transcription levels of Runx2, ALP, Col I, and OCN displayed that 1,25-dihydroxyvitamin D3 can considerably upregulate the transcription level of the osteogenic differentiation markers to promote the osteogenic differentiation of SCAPs, among which Runx2 serves as a marker factor for osteogenic differentiation of stem cells. The upregulation of the transcription level of this factor marks the entry of stem cells into the initial stage of bone formation [17]. As a phenotypic marker factor of osteoblasts, ALP's transcription level can reflect the activity of osteoblasts. The large amount of ALP expression after osteoblast differentiation of stem cells will promote the maturation and mineralization of extracellular matrix [18]. Mainly secreted by bone cells or chondrocytes, OCN is also a marker factor for osteogenic differentiation of stem cells, contributing to bone metabolism regulation [19]. Col I is one of the basic substances for bone formation and outer matrix mineralization [20–22]. The results of this study proved that 1,25-dihydroxyvitamin D_3_ can positively promote the osteogenic differentiation of SCAPs, and the expression of related osteogenic differentiation markers increased by upregulating the transcription levels of Runx2, ALP, OCN, and Col I. Combined with the above-mentioned discussion, 1,25-dihydroxyvitamin D_3_ can effectively enhance the adhesion, spreading, and proliferation of stem cells from apical papillary and can effectually promote the osteogenic differentiation of SCAPs and mineralization of extracellular matrix. This reminds us that 1,25-dihydroxyvitamin D_3_ might be used as a potential drug to induce the redevelopment of the root apex of young permanent teeth after trauma and promote the closure of the apical foramen.

The previous study compared the effects of 1,25-dihydroxyvitamin D_3_, basic fibroblast growth factor, and bone morphogenetic protein-2 on the osteogenic differentiation of stem cells. Compared with growth factors, 1,25-dihydroxyvitamin D_3_, as a clinical drug approved by the FDA, has a clear mechanism of action and a controllable concentration and dosage, especially for the special needs of oral clinical treatment [23]. 1,25-Dihydroxyvitamin D_3_ can be used as an additive to existing therapeutic drugs to enhance the therapeutic effect.

Thus, 1,25-dihydroxyvitamin D_3_ is expected to be used as an active ingredient in oral clinics. For example, as reported in previous studies [24, 25], 1,25-dihydroxyvitamin D_3_ can be used as an active drug and a biomaterial scaffold to form a bioengineered tooth root or root apex repair scaffold. Through the gradual degradation of the scaffold and the sustained release of 1,25-dihydroxyvitamin D3, root regeneration and root apex restoration are induced. Nevertheless, a further study is called for to delve into the exact mechanism of 1,25-dihydroxyvitamin D_3_ on cell proliferation and osteogenic differentiation so as to better guide clinical application and pharmaceutical preparations development.

## Figures and Tables

**Table 1:** Illustration of the sequences of the osteogenic-related genes, detected in the experiment. Primer sequence list.

Gene	Primer sequence (5′-3′)
*Col I*	F5′ CAGGCTGGTGTGATGGGATT 3′
	R5′ CCAAGGTCTCCAGGAACACC 3′
*OCN*	F5′ GCCCTGACTGCATTCTGCCTCT 3′
	R5′ TCACCACCTTACTGCCCTCCTG 3′
*ALP*	F5′ TATGTCTGGAACCGCACTGAAC 3′
	R5′ ACTAGCAAGAAGAAGCCTTTGG 3′
*Runx2*	F5′ ATCCAGCCACCTTCACTTACACC 3′
	R5′ GGGACCATTGGGAACTGATAGG 3′
*GAPDH*	F5′ CACCCGCGAGTACAACCTTC 3′
	R5′ CCCATACCCACCATCACACC 3′

## Data Availability

All data generated or used during the study appear in the article.

## Abbreviations

SCAPs: Stem cells from apical papilla
Runx2: Runt-related transcription factor 2
ALP: Alkaline phosphatase
Col I: Collagen I
OCN: Osteocalcin.
